# Supplementary material for: Trade-offs in cotton pest management: Seed treatments suppress pests but reduce the abundance of natural enemies in the arthropod community
Source: PLoS One. 2026 Apr 21;21(4):e0346422. doi: 10.1371/journal.pone.0346422 (PMC13098939; doi:10.1371/journal.pone.0346422)
Supplement: S1 Table — (PDF) [file pone.0346422.s001.pdf]

S1 Table. Mean sums of Cicadellidae and Miridae populations collected per plot using sweep nets

**2021**	**Cicadellidae**											
	**18 June**	**25 june**	**1 July**	**9 july**	**19 july**	**27 july**	**5 August**	**13 August**	**19 August**	**27 August**	**3 September**	**7 September**
**Untreated Control**	844±44.55c	898±105.74b	345±5.69b	170±33.41b	165±6.49b	328±12.77c	144±3.06b	103±3.06b	690±61.70b	966±22.52c	796±7.80c	1478±27.42b
**CLO**	353±15.41a	614±8.50a	181±2.52a	65±2.52a	86±4.62a	172±1.15a	85±6.36a	36±2.19a	140±1.15a	218±8.66a	325±1.53a	887±5.24a
**AMF**	673±13.87b	834±8.45ab	262±42.65ab	142±2.60ab	95±4.36a	200±2.89b	92±.73a	110±12.66b	230±2.52a	379±10.82b	386±2.31b	1530±6.81b
F	76,990	5,903	10,858	7,869	68,662	120,657	58,644	28,434	68,458	674,695	2880,070	462,622
P	0,000	0,038	0,010	0,021	0,000	0,000	0,000	0,001	0,000	0,000	0,000	0,000
**2021**	**Miridae**											
**Untreated Control**	55±3.53c	135±3.76c	121±2.08c	262±2.33c	339±3.84c	1297±14.98c	317±8.74c	413±12.00b	84±3.38b	67±2.65c	2±0.33a	1±0.33a
**CLO**	16±2.52a	2±0.58a	37±2.60a	37±2.19a	125±3.51a	423±2.73a	274±3.79b	323±11.61a	43±2.19a	0±0.33a	0±0.33a	0±0.33a
**AMF**	28±2.19b	91±1.20b	59±2.00b	228±3.76b	258±3.61b	660±2.08b	240±7.94a	293±2.33a	73±1.76b	39±1.73b	0±0.33a	1±0.33a
F	51,976	871,497	374,346	1820,717	875,604	2594,273	29,069	41,149	71,695	332,484	5,333	0,333
P	0.000	0,000	0,000	0,000	0,000	0,000	0,001	0,000	0,000	0,000	0,047	0,729

**2022**	**Cicadellidae**											
	**17 june**	**24 june**	**01 july**	**08 july**	**13 july**	**29 july**	**4 August**	**12 August**	**19August**	**26 August**	**2 September**	**9 September**
**Untreated Control**	995±12.90a	522±13.08a	521±23.03a	620±27.43a	604±19.00a	911±180.13a	195±3.06a	180±79.03a	205±5.03a	141±25.15a	104±8.74a	30±5.00a
**CLO**	497±166.01b	322±96.67a	139±31.13c	143±74.36b	163±63.13c	577±13.11a	94±15.52b	211±119.64a	26±7.21c	76±23.12a	62±3.79b	3±0.58b
**AMF**	729±48.79ab	519±100.92a	253±33.56b	361±2.60b	408±41.02b	682±39.95a	104±30.07b	243±28.54a	64±7.77b	83±12.66a	75±9.29b	21±7.37a
F	6,188	2,000	43,935	36,235	24,294	2,558	8,046	0,139	193,823	2,878	7,836	8,297
P	0,035	0,216	0,000	0,000	0,001	0,157	0,020	0,873	0,000	0,133	0,021	0,019
**2022**	**Miridae**											
**Untreated Control**	93±14.2a	127±9.7a	118±3.5a	298±49.7a	357±26.2a	849±73.2a	329±23.7a	435±6.2a	97±38.3a	65±2.1a	1±0.6a	0±0.0
**CLO**	63±6.0a	8±0.6c	35±4.9c	71±8.5b	57±9.7c	76±2.5c	203±74.0a	284±32.0b	41±3.2a	8±1.5b	0±0.0a	0±0.0
**AMF**	75±8.9a	86±5.1b	66±6.2b	212±15.9a	228±29.2b	294±19.1b	271±23.6a	379±87.5ab	62±2.0a	13±2.3b	0±0.0a	0±0.0
F	2,160	90,603	70,959	14,088	41,639	83,151	1,807	3,597	1,623	249,083	3,000	-
P	0,197	0,000	0,000	0,005	0,000	0,000	0,243	0,094	0,273	0,000	0,125	-

Column means within a year followed by the different letter are significantly different according to the ANOVA.
