# Supplementary material for: Trade-offs in cotton pest management: Seed treatments suppress pests but reduce the abundance of natural enemies in the arthropod community
Source: PLoS One. 2026 Apr 21;21(4):e0346422. doi: 10.1371/journal.pone.0346422 (PMC13098939; doi:10.1371/journal.pone.0346422)
Supplement: S2 Table — (PDF) [file pone.0346422.s002.pdf]

S2 Table. Mean (±SD) abundance of arthropod families (Miridae, Cicadellidae, Anthocoridae, Asillidae, Coccinellidae and Nabidae) under different seed treatments across sampling weeks in 2021 and 2022

Mean±SE						
	Cicadellidae			Miridae		
**2021**	**Control**	**Clothianidin**	**Azoxystrobin-Metalaxyl M**	**Control**	**Clothianidin**	**Azoxystrobin-Metalaxyl M**
**1**	844±45	354±15	673±14	55±3,53	16±2,52	28±2,19
**2**	899±106	614±9	834±8	135±3,76	2±0,58	91±1,20
**3**	345±6	181±3	263±43	121±2,08	37±2,60	59±2,00
**4**	170±33	65±3	142±3	262±2,33	37±2,19	228±3,76
**5**	165±6	86±5	95±4	339±3,84	125±3,51	258±3,61
**6**	328±13	172±1	200±3	1297±14,98	423±2,73	660±2,08
**7**	144±3	85±6	92±2	317±8,74	274±3,79	240±7,94
**8**	103±3	36±2	110±13	413±12,00	323±11,61	293±2,33
**9**	690±62	140±1	230±3	84±3,38	43±2,19	73±1,76
**10**	966±23	218±9	364±11	67±2,65	0±0,33	39±1,73
**11**	796±8	325±2	386±2	2±0,33	0±0,33	0±0,33
**12**	1478±27	888±5	1530±7	1±0,33	0±0,33	1±0,33
	Nabidae			Chrysopidae		
**2021**	**Control**	**Clothianidin**	**Azoxystrobin-Metalaxyl M**	**Control**	**Clothianidin**	**Azoxystrobin-Metalaxyl M**
**1**	2±0,33	2±0,33	1±0,33	2±0,33	0±0,33	2±0,33
**2**	6±0,58	1±0,33	3±0,00	3±0,67	2±0,00	3±0,33
**3**	2±0,33	2±0,58	2±0,33	1±0,33	0±0,33	1±0,33
**4**	3±0,67	1±0,33	1±0,33	0±0,00	0±0,00	0±0,00
**5**	1±0,00	1±0,33	1±0,00	0±0,00	0±0,00	0±0,00
**6**	2±0,33	1±0,67	1±0,67	3±0,00	3±0,33	2±0,33
**7**	1±0,00	1±0,00	1±0,33	4±0,33	2±0,58	2±0,33
**8**	0±0,00	0±0,00	0±0,00	3±0,33	1±0,00	3±0,33
**9**	2±0,33	1±0,00	2±0,00	6±0,58	2±0,67	4±0,67
**10**	0±0,00	0±0,33	0±0,33	7±0,58	4±0,58	7±0,67
**11**	0±0,33	0±0,33	0±0,33	6±0,33	6±0,58	6±0,33
**12**	2±0,33	1±0,67	0±0,00	6±0,33	6±0,33	5±0,33
	Coccinellidae			Asillidae		
**2021**	**Control**	**Clothianidin**	**Azoxystrobin-Metalaxyl M**	**Control**	**Clothianidin**	**Azoxystrobin-Metalaxyl M**
**1**	9±0,33	6±0,58	9±1,20	6±0,33	5±0,33	6±0,33
**2**	10±0,33	4±0,33	9±1,00	3±0,00	0±0,33	3±0,00
**3**	11±0,67	11±0,88	10±0,33	4±0,33	2±0,33	7±0,33
**4**	20±0,33	2±0,33	2±0,33	4±0,58	2±0,33	3±0,33
**5**	1±0,33	1±0,00	0±0,33	3±0,33	2±0,33	3±0,00
**6**	24±0,00	17±1,20	23±0,00	5±0,58	2±0,33	3±0,33
**7**	4±0,88	3±0,33	2±0,33	8±0,33	3±0,33	6±0,88
**8**	0±0,33	0±0,00	0±0,00	3±1,15	3±0,33	3±0,58
**9**	1±0,00	1±0,00	0±0,00	2±0,33	0±0,33	1±0,58
**10**	1±0,33	0±0,33	0±0,33	1±0,33	1±0,33	0±0,33
**11**	1±0,33	1±0,33	0±0,00	0±0,00	0±0,00	0±0,00
**12**	1±0,33	0±0,00	1±0,33	0±0,00	0±0,00	0±0,00
	Anthacoridae					
**2021**	**Control**	**Clothianidin**	**Azoxystrobin-Metalaxyl M**			
**1**	1±0,00	0±0,00	0±0,33			
**2**	2±0,58	0±0,33	1±0,58			
**3**	3±0,88	0±0,33	2±0,33			
**4**	5±1,45	1±0,67	2±0,67			
**5**	7±1,15	2±0,33	4±0,33			

**6**	9±0,88	5±0,33	4±0,58			
**7**	22±0,58	20±0,33	22±1,53			
**8**	13±3,28	5±0,33	8±0,88			
**9**	6±0,58	5±0,67	5±0,33			
**10**	3±0,33	1±0,33	1±0,19			
**11**	3±0,67	1±0,33	2±0,33			
**12**	2±0,33	1±0,33	1±0,33			
**Cicadellidae**				**Miridae**		
**2022**	**Control**	**Clothianidin**	**Azoxystrobin-Metalaxyl M**	**Control**	**Clothianidin**	**Azoxystrobin-Metalaxyl M**
**1**	995±13	497±166	729±49	93±14	63±6	75±9
**2**	522±13	322±97	519±101	127±10	8±1	86±5
**3**	521±23	139±31	253±34	118±4	34±5	66±6
**4**	620±27	143±74	142±3	298±50	71±9	212±16
**5**	604±19	163±63	408±41	357±26	57±10	228±29
**6**	911±180	577±13	682±40	849±73	76±3	294±19
**7**	195±3	94±16	104±30	329±24	203±74	271±24
**8**	180±79	211±120	243±29	435±6	284±32	479±88
**9**	205±5	26±7	64±8	97±38	41±3	62±2
**10**	141±25	76±23	83±13	65±2	8±2	13±2
**11**	104±9	62±4	75±9	1±1	0±0	0±0
**12**	30±5	1±1	21±7	0±0	0±0	0±0
**Nabidae**				**Chrysopidae**		
**2022**	**Control**	**Clothianidin**	**Azoxystrobin-Metalaxyl M**	**Control**	**Clothianidin**	**Azoxystrobin-Metalaxyl M**
**1**	2±0	2±1	1±1	3±1	1±0	3±1
**2**	6±1	1±0	3±0	5±0	0±0	3±2
**3**	2±0	2±1	2±0	3±0	1±0	0±0
**4**	3±1	1±0	1±0	1±1	1±1	0±0
**5**	1±0	1±0	1±0	2±1	0±0	1±0
**6**	2±0	1±1	1±1	3±0	1±0	1±0
**7**	1±0	1±0	1±0	3±1	0±0	0±0
**8**	0±0	0±0	0±0	7±1	4±1	3±0
**9**	2±0	1±0	2±0	6±1	1±1	1±0
**10**	0±0	0±0	0±0	4±2	0±0	3±0
**11**	0±0	0±0	0±0	5±1	2±1	3±1
**12**	2±0	1±1	0±0	2±1	3±1	0±0
**Coccinellidae**				**Asillidae**		
**2022**	**Control**	**Clothianidin**	**Azoxystrobin-Metalaxyl M**	**Control**	**Clothianidin**	**Azoxystrobin-Metalaxyl M**
**1**	1±1	0±0	0±0	5±0	0±0	2±0
**2**	2±1	0±0	1±0	3±0	0±0	2±0
**3**	0±0	0±0	0±0	3±1	1±0	2±1
**4**	3±0	0±0	1±1	6±1	1±0	1±0
**5**	1±0	0±0	0±0	5±1	1±1	3±0
**6**	2±0	0±0	0±0	4±1	2±1	4±0
**7**	0±0	0±0	0±0	6±1	1±0	1±0
**8**	2±0	1±0	1±0	1±0	1±0	0±0
**9**	2±1	0±0	0±0	5±1	3±0	1±0
**10**	0±0	0±0	0±0	8±1	4±1	6±1
**11**	0±0	0±0	0±0	0±0	0±0	0±0
**12**	0±0	0±0	0±0	4±1	1±0	0±0
**Anthacoridae**						
**2022**	**Control**	**Clothianidin**	**Azoxystrobin-Metalaxyl M**			
**1**	1±0	0±0	2±1			
**2**	1±0	0±0	1±1			
**3**	1±1	0±0	0±0			
**4**	7±1	2±2	4±2			
**5**	12±1	1±1	1±5			

**6**	17±5	2±1	7±1	
**7**	20±5	17±2	14±7	
**8**	14±3	4±2	4±2	
**9**	10±2	2±1	0±0	
**10**	0±3	1±1	1±0	
**11**	0±0	0±0	0±0	
**12**	0±0	0±0	0±0	
