# Supplementary figures and images for: Trade-offs in cotton pest management: Seed treatments suppress pests but reduce the abundance of natural enemies in the arthropod community
Source: PLoS One. 2026 Apr 21;21(4):e0346422. doi: 10.1371/journal.pone.0346422 (PMC13098939; doi:10.1371/journal.pone.0346422)

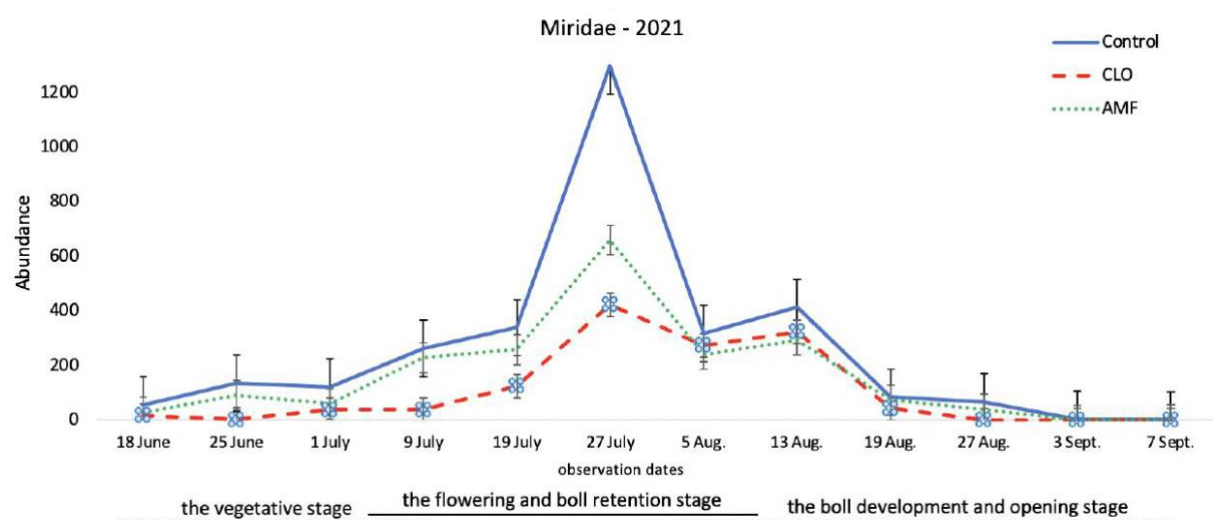

**S3 Fig. Impact of pesticide treatments on Miridae abundance during crop development stages in 2021**

Supplement: S3 Fig — (PDF) [file pone.0346422.s005.pdf]

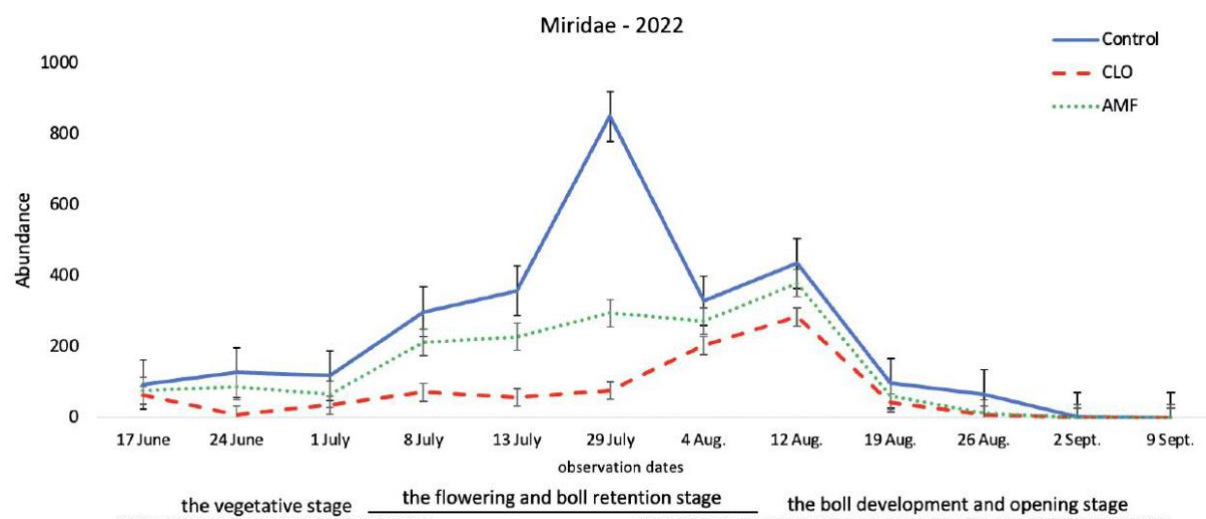

**S4 Fig. Impact of pesticide treatments on Miridae abundance during crop development stages in 2022**

Supplement: S4 Fig — (PDF) [file pone.0346422.s006.pdf]

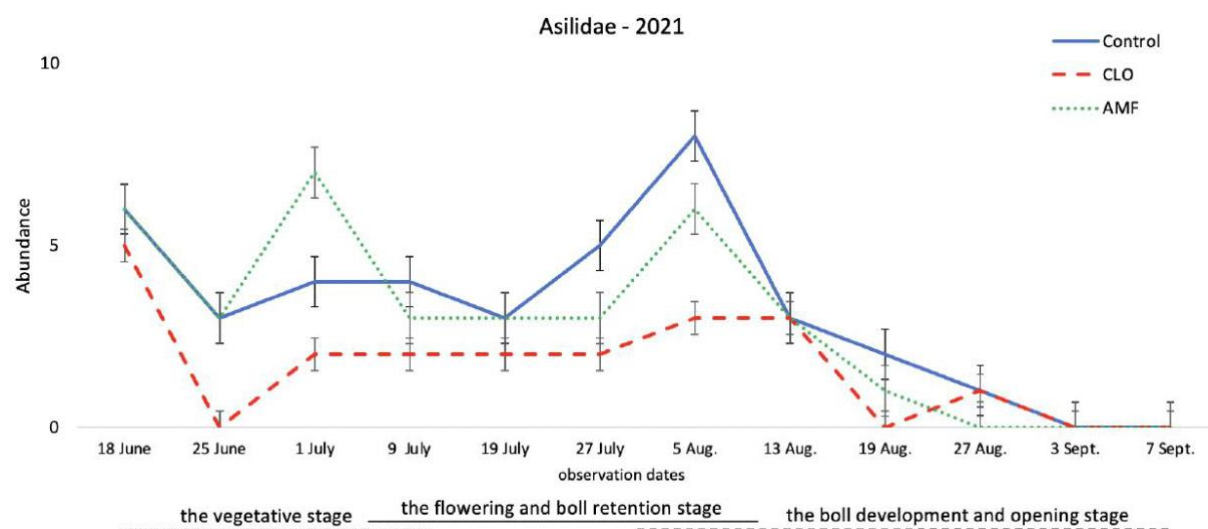

**S7 Fig. Impact of pesticide treatments on Asilidae abundance during crop development stages in 2021**

Supplement: S7 Fig — (PDF) [file pone.0346422.s009.pdf]

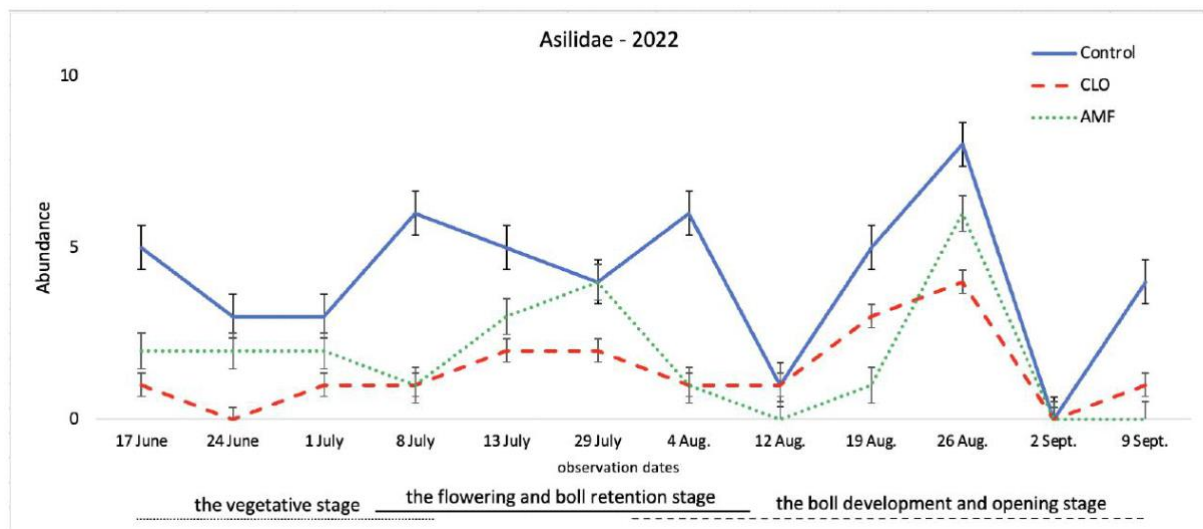

**S8 Fig. Impact of pesticide treatments on Asilidae abundance during crop development stages in 2022**

Supplement: S8 Fig — (PDF) [file pone.0346422.s010.pdf]

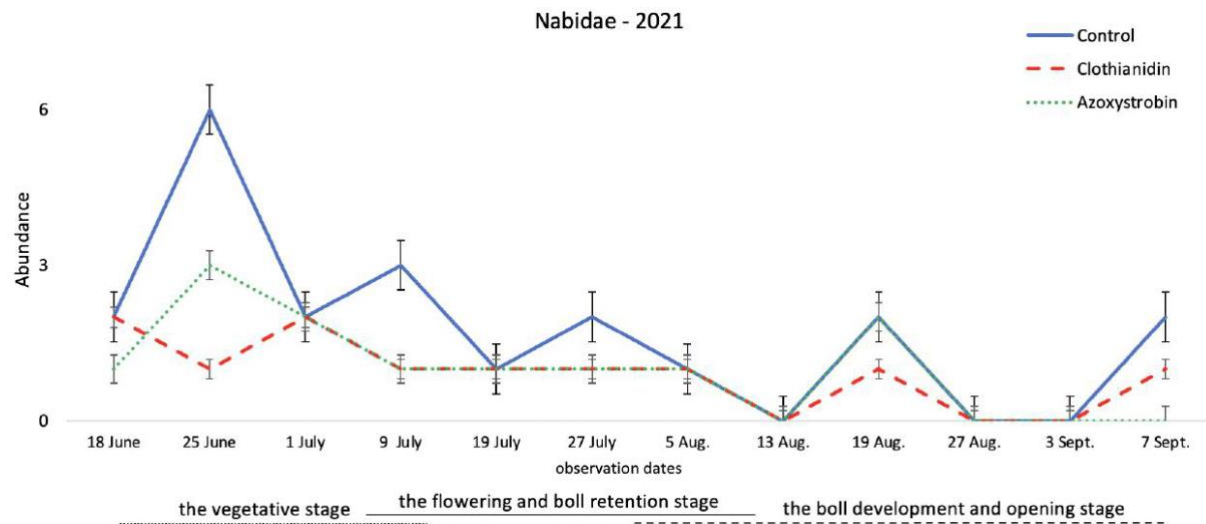

**S13 Fig. Impact of pesticide treatments on Nabidae abundance during crop development stages in 2021**

Supplement: S13 Fig — (PDF) [file pone.0346422.s015.pdf]

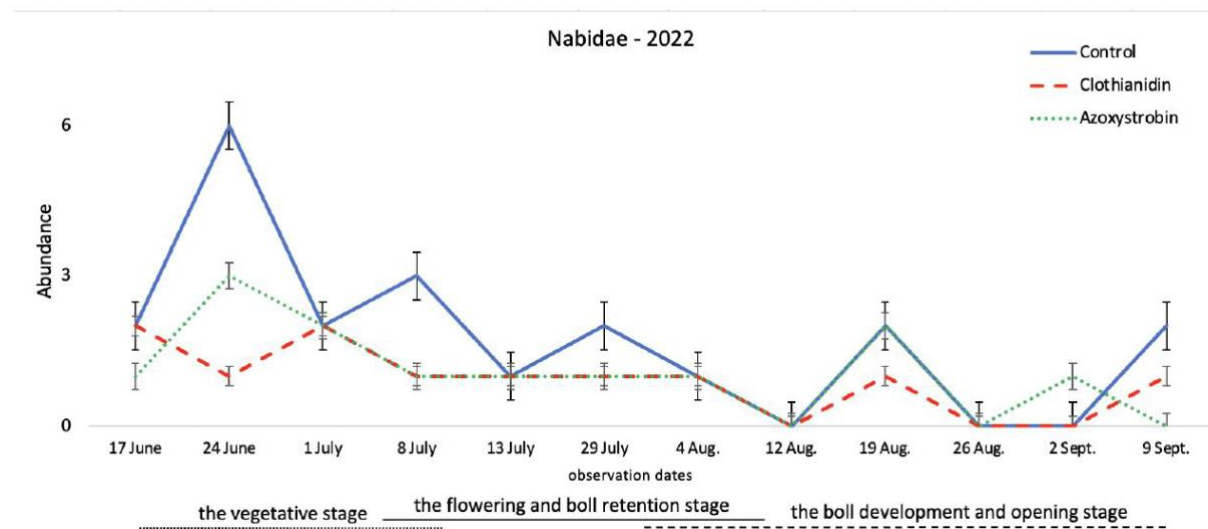

**S14 Fig. Impact of pesticide treatments on Nabidae abundance during crop development stages in 2022**

Supplement: S14 Fig — (PDF) [file pone.0346422.s016.pdf]
