# Supplementary material for: Trade-offs in cotton pest management: Seed treatments suppress pests but reduce the abundance of natural enemies in the arthropod community
Source: PLoS One. 2026 Apr 21;21(4):e0346422. doi: 10.1371/journal.pone.0346422 (PMC13098939; doi:10.1371/journal.pone.0346422)
Supplement: S12 Fig — (PDF) [file pone.0346422.s014.pdf]

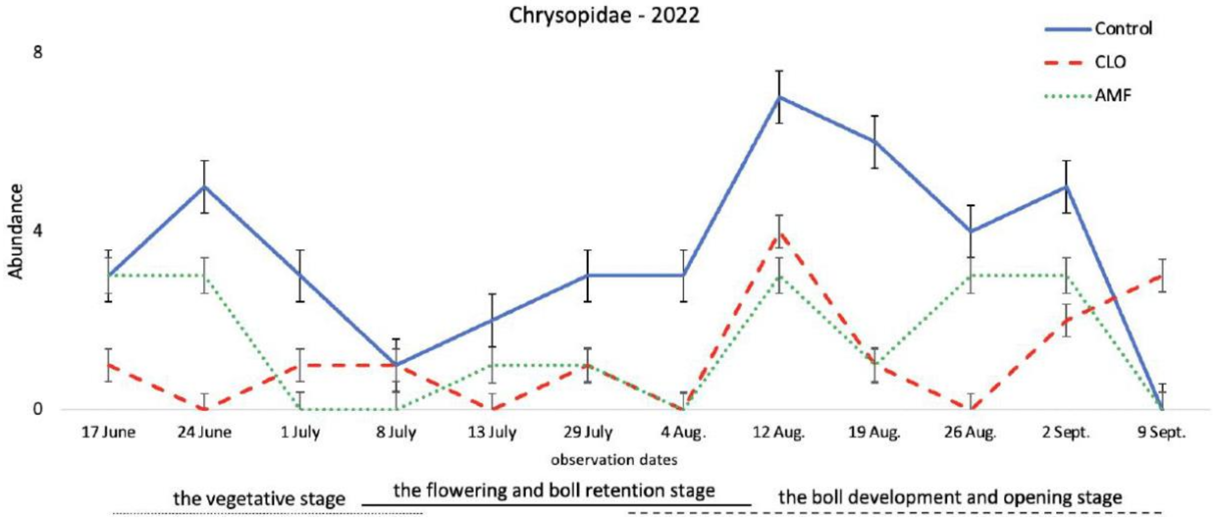


**S12 Fig. Impact of pesticide treatments on Chrysopidae abundance during crop development stages in 2022**
