# Supplementary material for: Trade-offs in cotton pest management: Seed treatments suppress pests but reduce the abundance of natural enemies in the arthropod community
Source: PLoS One. 2026 Apr 21;21(4):e0346422. doi: 10.1371/journal.pone.0346422 (PMC13098939; doi:10.1371/journal.pone.0346422)
Supplement: Attachment — Submitted filename: MYResponse to the reviewers comments.docx [file pone.0346422.s018.docx]

Answers to the reviewers comments

**View Letter**

| **Date:** | Feb 04 2026 12:16AM |
| --- | --- |
| **To:** | "Melis Yalçın" melisusluy@gmail.com |
| **From:** | "PLOS ONE" plosone@plos.org |
| **Subject:** | PLOS ONE Decision: Revision required [PONE-D-25-50468] |

PONE-D-25-50468

Balancing pest control and natural enemy conservation: Seed Treatment Effects in Cotton

PLOS One

Dear Dr. Yalçın,

Thank you for submitting your manuscript to PLOS ONE. After careful consideration, we feel that it has merit but does not fully meet PLOS ONE’s publication criteria as it currently stands. Therefore, we invite you to submit a revised version of the manuscript that addresses the points raised during the review process.

When revising your manuscript and as requested by reviewer 2, please give special consideration to (Abstract, supporting materials, double check figures/tables references and discussion flow). Also you should well describe in detail the sweeping methods (trapped insects and stages) in Mat&Met section.

Good luck

Please submit your revised manuscript by Mar 21 2026 11:59PM. If you will need more time than this to complete your revisions, please reply to this message or contact the journal office at [plosone@plos.org](mailto:plosone@plos.org). When you're ready to submit your revision, log on to https://www.editorialmanager.com/pone/ and select the 'Submissions Needing Revision' folder to locate your manuscript file.

Please include the following items when submitting your revised manuscript:

- A letter that responds to each point raised by the academic editor and reviewer(s). You should upload this letter as a separate file labeled 'Response to Reviewers'.
- A marked-up copy of your manuscript that highlights changes made to the original version. You should upload this as a separate file labeled 'Revised Manuscript with Track Changes'.
- An unmarked version of your revised paper without tracked changes. You should upload this as a separate file labeled 'Manuscript'.

If you would like to make changes to your financial disclosure, please include your updated statement in your cover letter. Guidelines for resubmitting your figure files are available below the reviewer comments at the end of this letter.

If applicable, we recommend that you deposit your laboratory protocols in protocols.io to enhance the reproducibility of your results. Protocols.io assigns your protocol its own identifier (DOI) so that it can be cited independently in the future. For instructions see: <https://journals.plos.org/plosone/s/submission-guidelines#loc-laboratory-protocols>. Additionally, PLOS ONE offers an option for publishing peer-reviewed Lab Protocol articles, which describe protocols hosted on protocols.io. Read more information on sharing protocols at <https://plos.org/protocols?utm_medium=editorial-email&utm_source=authorletters&utm_campaign=protocols>.

**Response to Reviewer:** Thank you for your suggestion regarding the deposition of laboratory protocols in protocols.io. I appreciate the opportunity to enhance methodological transparency and reproducibility.

However, as the procedures applied in my study follow established and previously published methodologies, I believe that additional protocol deposition is not necessary at this stage.

We look forward to receiving your revised manuscript.

Kind regards,

Rachid Bouharroud
Academic Editor
PLOS One

Journal Requirements:

When submitting your revision, we need you to address these additional requirements.

1. Please ensure that your manuscript meets PLOS ONE's style requirements, including those for file naming. The PLOS ONE style templates can be found at

https://journals.plos.org/plosone/s/file?id=wjVg/PLOSOne_formatting_sample_main_body.pdf and

<https://journals.plos.org/plosone/s/file?id=ba62/PLOSOne_formatting_sample_title_authors_affiliations.pdf>

**Response to Reviewer:** Thank you for your reminder regarding compliance with PLOS ONE’s style requirements. I confirm that the revised manuscript has been carefully checked and formatted in accordance with the journal’s guidelines, including file naming conventions and the use of the appropriate PLOS ONE style template.

2. Please ensure that you include a title page within your main document. We do appreciate that you have a title page document uploaded as a separate file, however, as per our author guidelines (http://journals.plos.org/plosone/s/submission-guidelines#loc-title-page) we do require this to be part of the manuscript file itself and not uploaded separately.

Could you therefore please include the title page into the beginning of your manuscript file itself, listing all authors and affiliations.

**Response to Reviewer:** Thank you for your clarification regarding the title page requirements. I have revised the submission accordingly and incorporated the title page at the beginning of the main manuscript file, including the author name and affiliation, in accordance with the PLOS ONE author guidelines. The manuscript has been re-uploaded with this modification.

3. Please ensure that you include a title page within your main document. You should list all authors and all affiliations as per our author instructions and clearly indicate the corresponding author.

**Response to Reviewer:** I would like to confirm that the title page has been prepared in accordance with the journal’s author guidelines. Author information and affiliation are included, and the corresponding author has been clearly indicated within the manuscript file.

4. Please include captions for your Supporting Information files at the end of your manuscript, and update any in-text citations to match accordingly. Please see our Supporting Information guidelines for more information: <http://journals.plos.org/plosone/s/supporting-information>.

**Response to Reviewer:** Thank you for your helpful comment regarding the Supporting Information files. In accordance with the journal’s guidelines, captions for all Supporting Information files have now been included at the end of the manuscript. In addition, all in-text citations have been carefully revised to ensure full consistency with the corresponding Supporting Information labels.

Furthermore, to improve clarity and conciseness of the manuscript, several references (Refs. 8, 9, 11, 13, 14, 15, 16, 17, 25, and 27) have been removed from the Introduction and Discussion sections. Accordingly, the reference list has been updated and renumbered, and all in-text citations have been revised to ensure consistency throughout the manuscript.

5. If the reviewer comments include a recommendation to cite specific previously published works, please review and evaluate these publications to determine whether they are relevant and should be cited. There is no requirement to cite these works unless the editor has indicated otherwise. 


[Note: HTML markup is below. Please do not edit.]

Reviewers' comments:

Reviewer's Responses to Questions

**Comments to the Author**

1. Is the manuscript technically sound, and do the data support the conclusions?

The manuscript must describe a technically sound piece of scientific research with data that supports the conclusions. Experiments must have been conducted rigorously, with appropriate controls, replication, and sample sizes. The conclusions must be drawn appropriately based on the data presented.

Reviewer #1: Partly

Reviewer #2: Yes

**Response to Reviewer:** The manuscript has been carefully revised to further strengthen its technical robustness and ensure that all conclusions are fully supported by the data presented.

Specifically, the following improvements have been made:

- The experimental design, including controls, replication, and sampling procedures, has been clarified in the Materials and Methods section to ensure transparency and reproducibility.
- Statistical analyses have been revised and reported consistently in accordance with journal guidelines (e.g., F-values, degrees of freedom, and p-values), ensuring clarity and accuracy.
- Seasonal mean abundances (± SEM) have been calculated and presented in a newly structured table to allow clearer comparison among treatments, as recommended.
- Data presentation has been simplified, with detailed temporal trends moved to supplementary materials, improving readability and interpretation of results.
- The Results and Discussion sections have been refined to ensure that all conclusions are directly supported by the data, avoiding overinterpretation.

These revisions have substantially improved the clarity, rigor, and consistency of the manuscript. Therefore, the study now fully meets the criteria for technical soundness, with conclusions appropriately supported by the presented data.

2. Has the statistical analysis been performed appropriately and rigorously?

Reviewer #1: I Don't Know

Reviewer #2: Yes

**Response to Reviewer:** The statistical analysis has been carefully revised and clarified throughout the manuscript to ensure transparency and reproducibility.

To address potential ambiguity, the following improvements have been made:

- The statistical methods have been described in greater detail in the Statistical Analysis section, including the specific models used, assumptions tested, and post-hoc procedures applied.
- Repeated-measures ANOVA has been clearly specified as the primary analytical approach for evaluating treatment effects over time.
- All statistical results are now reported consistently in accordance with journal guidelines (e.g., F-values with exact p-values).
- Post-hoc comparisons (Tukey’s HSD) are explicitly indicated, and their application is clearly explained.
- Data presentation has been improved by including seasonal mean values (± SEM), allowing clearer interpretation of treatment effects.

These revisions ensure that the statistical analyses are both rigorous and clearly presented. The uncertainty expressed by Reviewer #1 is likely due to insufficient detail in the previous version, which has now been fully addressed.

3. Have the authors made all data underlying the findings in their manuscript fully available?

The [PLOS Data policy](http://www.plosone.org/static/policies.action#sharing) requires authors to make all data underlying the findings described in their manuscript fully available without restriction, with rare exception (please refer to the Data Availability Statement in the manuscript PDF file). The data should be provided as part of the manuscript or its supporting information, or deposited to a public repository. For example, in addition to summary statistics, the data points behind means, medians and variance measures should be available. If there are restrictions on publicly sharing data—e.g. participant privacy or use of data from a third party—those must be specified.

Reviewer #1: Yes

Reviewer #2: Yes

4. Is the manuscript presented in an intelligible fashion and written in standard English?

PLOS ONE does not copyedit accepted manuscripts, so the language in submitted articles must be clear, correct, and unambiguous. Any typographical or grammatical errors should be corrected at revision, so please note any specific errors here.

Reviewer #1: No

Reviewer #2: Yes

**Response to Reviewer:** The manuscript has been thoroughly revised to improve clarity, readability, and overall language quality.

5. Review Comments to the Author

Please use the space provided to explain your answers to the questions above. You may also include additional comments for the author, including concerns about dual publication, research ethics, or publication ethics. (Please upload your review as an attachment if it exceeds 20,000 characters)

Reviewer #1: Introduction is fairly long. A number of studies are cited, indicating a lot of literature already available on this topic. What are objectives of this study needs to be enlisted and introduction should be shaped according to them to build argument by identifying gaps rather than reviewing earlier literature.

**Response to Reviewer:**
Thank you for this valuable and constructive comment. The Introduction has been carefully revised and substantially shortened to improve focus and clarity. In particular, the section has been restructured to better highlight the research gap and to clearly state the objectives of the study, thereby strengthening the overall rationale.

To address this concern, the following revisions have been made:

- Lines 52–53 and 59–64 have been removed to eliminate redundant background information.
- The literature presented in Lines 84–89 has been revised to focus specifically on neonicotinoids in relation to clothianidin, and the references have been updated accordingly.
- Lines 103–148 have been removed to reduce excessive literature review and improve conciseness.
- The Introduction has been streamlined and reduced to 559 words.

As a result, the revised Introduction is now more concise, better aligned with the study objectives, and more clearly structured around the identified research gap.

L102: For the experimental design, three replicates were run following a randomized complete block design. In a small-scale study of this kind, different field as replicates may be more appropriate to include variation as true replicates.

**Response to Reviewer:** Thank you for this valuable suggestion. I agree that conducting similar studies across different fields would provide broader insight and increase environmental variability. However, the present study was intentionally conducted within the same field under uniform conditions to minimize environmental heterogeneity and ensure comparability among treatments. This approach allowed us to focus specifically on treatment effects. Moreover, previous multi-site field studies and meta-analyses have reported consistent patterns regarding the effects of neonicotinoid seed treatments on natural enemy communities across different geographic locations [Douglas and Tooker, 2016; Saeed and Razaq, 2016], suggesting that major outcome differences among fields may be limited.

Douglas MR, Tooker JF. Meta-analysis reveals that seed-applied neonicotinoids and pyrethroids have similar negative effects on abundance of arthropod natural enemies. PeerJ. 2016 Dec 7;4:e2776. doi: 10.7717/peerj.2776. PMID: 27957400; PMCID: PMC5147019.

Saeed R, Razaq M, Hardy IC. Impact of neonicotinoid seed treatment of cotton on the cotton leafhopper, Amrasca devastans (Hemiptera: Cicadellidae), and its natural enemies. Pest Manag Sci. 2016 Jun;72(6):1260-7. doi: 10.1002/ps.4146. Epub 2015 Oct 5. PMID: 26436945.

L119: Sweep nests were used to assess abundance. If this method was used against flying adults or also when larvae or immobile or less immobile stages were present, needs clarity on this. how much time was spent per sweep.

**Response to Reviewer:** Sweep net sampling was used to assess the abundance of mobile foliar-dwelling arthropods present in the plant canopy. For hemimetabolous groups, including Miridae, Cicadellidae, Nabidae and Anthocoridae, both nymphs and adults captured by sweep netting were included in the counts because these taxa undergo incomplete metamorphosis and their nymphal stages are active and mobile within the canopy. In contrast, for holometabolous groups such as Chrysopidae and Asilidae, only adult individuals were recorded, as their larval stages occur in different microhabitats (e.g., soil or concealed substrates) and are not effectively sampled using sweep netting (Line173-179).

Sampling was conducted for twelve consecutive weeks during the cotton growing seasons of 2021 and 2022 (June–September). During each sampling event, 50 sweeps were performed per plot along a straight transect through the center of each plot. The experiment consisted of three replicates arranged in a randomized complete block design. Sweep netting was carried out at least 100 m from the field edge to minimize potential edge effects. The same standardized sampling protocol (50 sweeps per replicate per sampling date) was consistently applied across both years and treatments.

Results: Seasonal counts can be compared among treatments at the end of season. Line graphs can be made supplementary and give their seasonal means comparison, results are too long and hard to follow under current data representation. Also provide information on dominant species of pests and predators.

**Response to Reviewer:** Seasonal mean abundances (± SEM) for Miridae and Cicadellidae were calculated for each treatment and year and are now presented in a newly developed Table 1. Treatment effects were analysed using repeated measures ANOVA followed by Tukey’s HSD test, and significant differences among treatments are indicated by different letters within each year.

Four line graphs previously included in the main manuscript have been removed from the Results section and are now presented as Supplementary Figures (Figures S1–S4). This significantly reduces redundancy and improves readability. The main text now focuses on seasonal mean comparisons, while the figures illustrating weekly population dynamics remain available in the Supplementary Material for reference.

Dominant pest and predator groups have been explicitly stated. Cicadellidae and Miridae are now clearly identified as the dominant sucking pest groups across both years. Similarly, the dominant predator taxa have been explicitly indicated in the revised text. This revision improves the ecological interpretation of the results and addresses the reviewer’s concern.

Discussion: What authors find new in this study and discuss the way forward. What are the limitation of this study and possible research direction?

**Response to Reviewer:** Thank you for this valuable suggestion. The present study provides novel field-based evidence that seed-applied systemic pesticides can influence not only target sucking pest populations but also the abundance and diversity of associated predator communities in cotton agroecosystems. While previous studies have primarily focused on the efficacy of seed treatments against early-season pests(Douglas and Tooker, 2015; Jeschke et al., 2011), their broader ecological consequences at the community level remain poorly understood under field conditions. By simultaneously evaluating the seasonal dynamics of dominant sucking pests, such as Cicadellidae and Miridae, alongside key predator taxa, this study demonstrates that seed-applied clothianidin and fungicide-based treatments may exert cascading effects on arthropod community structure beyond their intended targets. These findings contribute to a more comprehensive understanding of how prophylactic seed treatments shape multitrophic interactions in cotton systems and highlight the importance of considering non-target ecological responses when assessing the sustainability of systemic pesticide applications.

The Discussion section has been revised to include a forward-looking perspective on the implications of our findings for sustainable pest management in cotton agroecosystems. Specifically, the revised text now highlights how seed-applied systemic pesticides may influence multitrophic arthropod interactions beyond their target pest species and emphasizes the importance of integrating non-target ecological effects into pest management decision-making frameworks. In addition, the potential role of community-level monitoring in improving the ecological sustainability of prophylactic seed treatments has been discussed. These additions are now incorporated in the revised Discussion section (Line 499-504, Line 523-531)

A paragraph outlining the main limitations of the present study and potential future research directions has been added to the Conclusion section. Specifically, the revised text now acknowledges the limited temporal scale of the two-year field study and the absence of species-specific and sublethal assessments. In addition, the need for long-term and species-level investigations to better understand the ecological consequences of seed-applied systemic pesticides on multitrophic arthropod interactions has been discussed(Line 545-551).

Reviewer #2: This two-year field study provides a valuable community-level assessment of the impact of two common seed treatments—clothianidin (CLO, insecticide) and azoxystrobin + metalaxyl-m + fludioxonil (AMF, fungicide combination)—on arthropod pests and their natural enemies in a cotton agroecosystem. The research addresses an important gap by moving beyond single-species assessments to evaluate whole-community dynamics, which is crucial for developing sustainable Integrated Pest Management (IPM) strategies.

Key Strengths of the Manuscript:

Strong Experimental Design: A two-year, replicated field trial with an untreated control provides robust, ecologically relevant data.

Comprehensive Community Analysis: The use of Principal Response Curve (PRC) analysis to synthesize community-level effects is a significant methodological strength, clearly visualizing how entire arthropod assemblages shift in response to treatments over time.

Important Findings: The study convincingly demonstrates that both CLO and AMF seed treatments significantly reduce the abundance of key natural enemy families (e.g., Nabidae, Chrysopidae, Coccinellidae) while also suppressing pest populations like Cicadellidae. The documented negative correlation between pest and predator suppression highlights a critical trade-off in pest management.

Clear Conclusions: The conclusions are well-supported by the data, emphasizing that seed treatments are not "safe" for natural enemies and calling for more judicious, need-based use within IPM frameworks.

Suggestions for Revision and Clarification:

Title Refinement: The current title ("Balancing pest control and natural enemy conservation: Seed Treatment Effects in Cotton") is slightly generic. Consider making it more specific to your key finding, e.g., "Trade-offs in cotton pest management: Seed treatments suppress pests but reduce natural enemy abundance in the arthropod community."

**Response to Reviewer:** Thank you for this helpful suggestion. The title has been revised to more clearly reflect the key findings of the study, particularly the trade-offs observed between pest suppression and the reduction in natural enemy abundance within the arthropod community, as recommended.

Abstract Enhancement: The abstract should more clearly state the main takeaway. Explicitly mention that your study found a significant reduction in predator abundance alongside pest suppression, creating a potential trade-off. Briefly specify the two treatments used.

**Response to Reviewer:** Thank you for this helpful suggestion. The Abstract has been revised to more clearly highlight the main findings of the study, including the observed reduction in predator abundance alongside pest suppression, indicating a potential trade-off in cotton pest management. In addition, the two seed treatment applications evaluated in the study, clothianidin (CLO) and the AMF fungicide mixture, are now explicitly stated(Line 13-28)

Results Presentation: The text frequently refers to figures and tables (e.g., Fig 1, S1 Table) that are not included in the provided text file. Ensure all in-text citations have corresponding, clearly labeled items in the submitted manuscript.

**Response to Reviewer::** Thank you for this comment. All figures and tables and supplementary figures and tables referenced in the text have been carefully reviewed to ensure that each in-text citation corresponds to a clearly labeled figure or table in the submitted manuscript and Supporting Information files.

Discussion Flow: The discussion is comprehensive but could be more tightly structured. Consider subheadings (e.g., "Effects on Pest Communities," "Effects on Predator Communities," "Community-Level Implications") to improve readability and guide the reader through your interpretations.

**Response to Reviewer:** Thank you for this helpful suggestion. The Discussion section has been reorganized using clearly defined subheadings to improve readability and guide the reader through the interpretation of the results.

Clarity on "Neonicotinoid" Terminology: Clothianidin is a neonicotinoid. The introduction and discussion mention neonicotinoids broadly but could more explicitly frame CLO within this well-known class from the outset, as their non-target effects are a major point of discussion.

**Response to Reviewer:**
Thank you for this helpful comment. Clothianidin has been explicitly identified as a neonicotinoid insecticide in the revised manuscript. In addition, the examples provided in the Introduction primarily refer to studies involving clothianidin and other neonicotinoid compounds to clearly situate CLO within this well-known chemical class. Likewise, the active ingredients discussed in the Discussion section (e.g., thiamethoxam and imidacloprid) also belong to the neonicotinoid group, which was used to contextualize the non-target effects reported in the literature. To further improve clarity, the classification of clothianidin as a neonicotinoid has now been explicitly stated in the relevant sections of the manuscript.

Statistical Reporting Consistency: Ensure that all statistical results (F-values, degrees of freedom, p-values) are reported consistently (e.g., F(1, 2) = 5.90, p = 0.038).

**Response to Reviewer:**
Thank you for this helpful suggestion. The reporting of statistical results has been carefully revised throughout the manuscript. All F-values and p-values have now been reformatted to ensure consistency and to comply with the reporting style recommended by PLOS ONE (e.g., F= value, p = value). These changes have been applied consistently across the Results section and corresponding tables where applicable.

Data Availability Statement: The statement "The data supporting this study's findings are available in this article's supplementary material" is good, but PLOS ONE requires that the data be fully available without restriction. Double-check that all raw data underlying the figures and analyses are indeed included in the submitted Supporting Information files.

**Response to Reviewer:**
Thank you for this important comment. All underlying raw data supporting the results presented in this study are available in the Supporting Information files, in accordance with PLOS ONE data availability requirements.

Specifically, the raw dataset is provided in S15 Table, which includes mean (± SD) abundance values for arthropod families (Miridae, Cicadellidae, Anthocoridae, Asilidae, Coccinellidae, and Nabidae) across all sampling weeks in 2021 and 2022 under different seed treatments.

This dataset contains the full set of observations used in the analyses and figures, ensuring that all results can be independently verified and reproduced without restriction.

6. PLOS authors have the option to publish the peer review history of their article ([what does this mean?](https://journals.plos.org/plosone/s/editorial-and-peer-review-process#loc-peer-review-history)). If published, this will include your full peer review and any attached files.


If you choose “no”, your identity will remain anonymous but your review may still be made public.


**Do you want your identity to be public for this peer review?** For information about this choice, including consent withdrawal, please see our [Privacy Policy](https://www.plos.org/privacy-policy).

Reviewer #1: No

Reviewer #2: No

[NOTE: If reviewer comments were submitted as an attachment file, they will be attached to this email and accessible via the submission site. Please log into your account, locate the manuscript record, and check for the action link "View Attachments". If this link does not appear, there are no attachment files.]

To ensure your figures meet our technical requirements, please review our figure guidelines: https://journals.plos.org/plosone/s/figures

You may also use PLOS’s free figure tool, NAAS, to help you prepare publication quality figures: https://journals.plos.org/plosone/s/figures#loc-tools-for-figure-preparation.

NAAS will assess whether your figures meet our technical requirements by comparing each figure against our figure specifications.

*In compliance with data protection regulations, you may request that we remove your personal registration details at any time.*  [*(Remove my information/details)*](https://www.editorialmanager.com/pone/login.asp?a=r)*. Please contact the publication office if you have any questions.*
